# Ferroptosis in Doxorubicin-Induced Cardiotoxicity: From Molecular Mechanisms to Therapeutic Strategies and Clinical Management Paradigms

**DOI:** 10.31083/RCM46166

**Published:** 2026-06-17

**Authors:** Peipei Zhang, Zilong Wu, Xiaoying Pan, Xinyi Chen, Weinian Xia, Zhuan Peng, Changyu Zhou, Jie Xiang, Li Wang, Dezhong Li, Kai Luo, Chuying Huang

**Affiliations:** ^1^Hubei Key Laboratory for Translational Research in Traditional Chinese Medicine, The Central Hospital of Enshi Tujia and Miao Autonomous Prefecture, Hubei Minzu University, 445000 Enshi, Hubei, China; ^2^College of Biological and Food Engineering, Hubei Minzu University, 445000 Enshi, Hubei, China; ^3^School of Public Health, Xiamen University, 361100 Xiamen, Fujian, China; ^4^Cardiovascular Disease Center, The Central Hospital of Enshi Tujia and Miao Autonomous Prefecture, Enshi Clinical College of Wuhan University, 445000 Enshi, Hubei, China

**Keywords:** ferroptosis, doxorubicin, cardiotoxicity, disease management, biomarkers

## Abstract

Despite substantial advances in oncology and cardiovascular research, the clinical use of doxorubicin (DOX) remains constrained by significant cardiotoxicity; meanwhile, ferroptosis is now recognized as a central mechanism underlying DOX-induced cardiotoxicity (DIC). DOX disrupts myocardial iron homeostasis, impairs key antioxidant defense systems, and induces lipid metabolic reprogramming that promotes the accumulation of peroxidation-prone substrates, driving excessive lipid peroxidation and ferroptotic cell death. Ferroptosis also closely interacts with apoptosis, pyroptosis, and related pathways, forming a synergistic cell death network characterized by PANoptosis, which exacerbates cardiac injury. An integrated strategy encompassing primary prevention, targeted inhibition of core ferroptotic pathways, and systemic cardiac repair has emerged, which includes cardiac-targeted nanodelivery systems, selective inhibitors of key regulators such as glutathione peroxidase 4 (GPX4), ferroptosis suppressor protein 1 (FSP1), and acyl-CoA synthetase long-chain family member 4, as well as pharmacological activation of endogenous cardioprotective pathways. Clinical monitoring is also evolving from conventional cardiac functional indices toward early-warning approaches that incorporate ferroptosis-related circulating biomarkers, such as stress products, sensitive imaging metrics, including global longitudinal strain (GLS), and artificial intelligence-assisted analyses. Therefore, this review provides a systematic and comprehensive synthesis of recent advances in the molecular mechanisms, therapeutic interventions, and clinical management frameworks of ferroptosis in DIC, aiming to deepen understanding of the role of this process in the pathogenesis and treatment of DOX-induced cardiac injury.

## 1. Introduction

Doxorubicin (DOX) is a highly effective anthracycline chemotherapeutic agent widely used in the treatment of both solid tumors and hematological malignancies [1,2]. However, its pronounced cardiotoxicity, manifesting as arrhythmias, irreversible ventricular dysfunction, and heart failure, represents a major dose-limiting complication that substantially compromises long-term survival and quality of life in cancer patients [3,4]. The pathogenesis of DOX-induced cardiotoxicity (DIC) is closely linked to topoisomerase II inhibition, which initiates upstream cellular stress responses, including oxidative stress and calcium overload, ultimately resulting in mitochondrial injury. These pathological alterations converge to activate multiple regulated cell death pathways, including apoptosis, pyroptosis, and autophagy. In recent years, ferroptosis, an iron-dependent form of regulated cell death driven by lipid peroxidation, has emerged as a critical contributor to the development of DIC [5].

DOX chelates Fe^3+^ to form complexes that promote excessive hydroxyl radical generation through the Fenton reaction and suppress the activity of glutathione peroxidase 4 (GPX4), accelerating mitochondrial lipid peroxidation and triggering mitochondria-dependent ferroptosis [6]. Ferroptosis is primarily induced by iron overload, which overwhelms cellular antioxidant defenses, and is tightly regulated by lipid metabolic pathways, iron homeostasis, and redox balance, rendering it highly relevant to the pathophysiology of DIC [7]. Beyond DIC, accumulating evidence indicates that ferroptosis also plays a vital role in the initiation and progression of a broad spectrum of cardiovascular diseases [8].

At present, clinical management of DIC relies mainly on conventional heart failure therapies, including β-blockers, angiotensin-converting enzyme inhibitors, and angiotensin II receptor blockers. Similarly, several natural compounds and pharmacological agents, such as luteolin, metformin, and melatonin, have been reported to exert cardioprotective effects [9]. Dexrazoxane is currently the only approved prophylactic agent for anthracycline-induced cardiotoxicity; it mitigates superoxide radical formation by converting to an open-ring structure that chelates free iron or DOX–iron complexes, reducing both the incidence and severity of cardiac injury [10]. However, the overall effectiveness of all these preventive and therapeutic strategies remains suboptimal, continuing to limit the broader clinical use of DOX [11]. As a result, there is an urgent need to develop safe and effective cardioprotective interventions for patients receiving DOX-based chemotherapy. In this context, elucidating the intricate interplay between DIC and ferroptosis and identifying ferroptosis-targeted cardioprotective agents with favorable safety profiles and translational potential are of considerable scientific and clinical significance.

## 2. Molecular Mechanisms of Ferroptosis in DIC

**Fig. 1.**
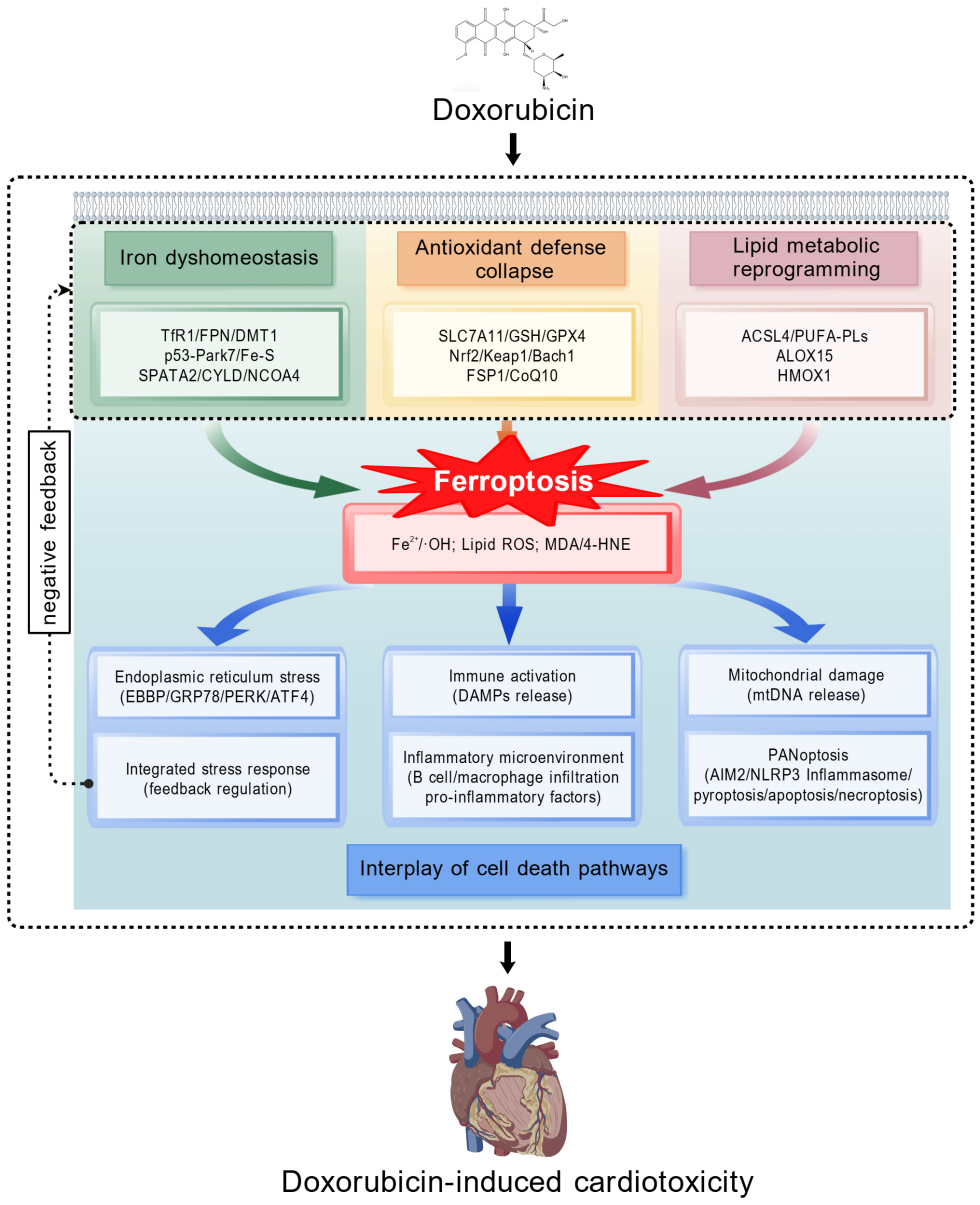
**Molecular mechanisms of ferroptosis in DIC**. This schematic summarizes the tripartite network mechanism through which doxorubicin (DOX) induces cardiomyocyte ferroptosis. At the primary level, DOX disrupts the homeostasis of iron metabolism, antioxidant defenses, and lipid metabolism, which converge to trigger lethal lipid peroxidation (the execution of ferroptosis). At the secondary level, ferroptosis acts as a pathogenic hub: it instigates mitochondrial damage, endoplasmic reticulum stress, and the release of damage signals, which in turn activate PANoptosis, the integrated stress response, and immune inflammation, respectively, forming an interactive amplification network. At the tertiary level, these cascades collectively lead to cardiomyocyte death, fibrosis, and cardiac dysfunction. TfR1, transferrin receptor 1; FPN, ferroportin; DMT1, divalent metal transporter 1; Fe–S, iron–sulfur clusters; SPATA2, spermatogenesis-associated protein 2; NCOA4, nuclear receptor coactivator 4; SLC7A11, solute carrier family 7 member 11; GSH, glutathione; GPX4, glutathione peroxidase 4; Nrf2, nuclear factor erythroid 2-related factor 2; Keap1, kelch-like ECH-associated protein 1; Bach1, BTB and CNC homology 1; FSP1, ferroptosis suppressor protein 1; CoQ10, coenzyme Q10; ACSL4, acyl-CoA synthetase long-chain family member 4; PUFA-PLs, polyunsaturated fatty acid-containing phospholipids; ALOX15, 15-lipoxygenase-1; HMOX1, heme oxygenase-1; OH, hydroxyl radical; ROS, reactive oxygen species; MDA, malondialdehyde; 4-HNE, 4-hydroxy-2-nonenal; EBBP, estrogen-responsive B-box protein; GRP78, glucose-regulated protein 78; PERK, protein kinase RNA-like endoplasmic reticulum kinase; ATF4, activating transcription factor 4; DAMPs, damage-associated molecular patterns; mtDNA, mitochondrial DNA; AIM2, absent in melanoma 2; NLRP3, NOD-like receptor family pyrin domain associated protein 3; CYLD, cylindromatosis; DIC, DOX-induced cardiotoxicity. (created with https://BioGDP.com)

Ferroptosis has been recognized as a central execution mechanism in DIC, resulting from a profound breakdown of cellular homeostasis, with its molecular pathways summarized in Fig. 1. DOX induces cardiomyocyte injury through coordinated disruption of three critical homeostatic systems, iron metabolism, antioxidant defenses, and lipid metabolism, while simultaneously activating other regulated cell-death programs, including apoptosis, necroptosis, and pyroptosis. Increasing evidence indicates substantial crosstalk and synergistic interactions among these pathways, giving rise to a highly integrated, multi-layered cell-death network. Therefore, this section systematically delineates how DOX compromises key regulatory nodes, precipitates widespread failure of cellular defense mechanisms, and ultimately drives ferroptosis-centered, multi-modal cardiomyocyte death.

### 2.1 Iron Dyshomeostasis

In DIC, iron overload arises not simply from enhanced iron uptake but from a disease-specific disruption of the entire “uptake–storage–export” regulatory axis. Unlike other pathological contexts, DOX interferes with the central regulatory protein *Park7/DJ-1* by promoting its ubiquitination and proteasomal degradation, which, in turn, destabilizes iron–sulfur clusters that are essential for cellular iron sensing and metabolic control [12]. This disruption not only compromises mitochondrial function but also aberrantly activates iron regulatory protein 1, leading to inappropriate upregulation of transferrin receptor 1 and initiating iron metabolic imbalance in DIC. DOX further exploits the autophagic degradation machinery by stabilizing nuclear receptor coactivator 4 (*NCOA4*), inducing excessive ferritinophagy. This process accelerates lysosomal degradation of ferritin, the principal intracellular iron storage complex, resulting in a substantial release of labile iron ions [13]. Such forced iron mobilization distinguishes the “active” iron overload observed in DIC from iron accumulation caused by simple iron excess. Similarly, DOX suppresses the activity of ferroportin, the sole known cellular iron exporter. Pharmacological activation of the nuclear factor erythroid 2–related factor 2 (*Nrf2*) pathway, which restores ferroportin expression, confers significant cardioprotection [14], further underscoring the pathogenic contribution of impaired iron efflux in DIC.

### 2.2 Antioxidant Defense Collapse

Under conditions of DOX-induced iron overload and oxidative stress, the cardiomyocyte antioxidant defense system undergoes progressive, multi-tiered functional failure, with endogenous compensatory responses generally insufficient to maintain redox homeostasis. In DIC, impairment of the GPX4 axis occurs through a dual mechanism. DOX directly inhibits the cystine transporter solute carrier family 7 member 11 (SLC7A11), resulting in depletion of substrates required for reduced glutathione (GSH) synthesis [15]. Similarly, DOX suppresses nuclear translocation and transcriptional activity of the master antioxidant regulator *Nrf2* through epigenetic mechanisms, including protein arginine methyltransferase 4-mediated modifications, reducing GPX4 expression [16]. This simultaneous loss of both enzymatic substrate and catalytic capacity creates a pronounced vulnerability within the antioxidant defense network.

The GSH-independent ferroptosis suppressor protein 1 (FSP1)–mediated ubiquinone–ubiquinol antioxidant system is also disrupted in DIC myocardium, as reflected by decreased FSP1 protein levels and enhanced ubiquitin-dependent degradation [17]. Upstream regulatory pathways are similarly perturbed by DOX, including competitive inhibition of *Nrf2* by the transcriptional repressor BTB and CNC homology 1 (Bach1) [18] and derepression of the pro-death c-Jun N-terminal kinase signaling pathway following the loss of GSH S-transferase P1 activity [19]. Although cardiomyocytes can inducibly generate endogenous protective mediators, such as prostaglandin E2, to activate limited cytoprotective signaling [20], these adaptive responses are insufficient to restore global antioxidant capacity during sustained DOX exposure.

The therapeutic efficacy of targeting these antioxidant pathways depends heavily on the specific metabolic context imposed by DOX. For example, activation of transcription factor EB to enhance autophagy under DOX stress, as observed with alternate-day fasting, paradoxically aggravated cardiotoxicity [21], underscoring that additional metabolic perturbations in an already destabilized system may be detrimental rather than protective.

### 2.3 Lipid Metabolic Reprogramming

DOX actively remodels lipid metabolism to generate a membrane environment enriched in oxidation-prone substrates while simultaneously enhancing the efficiency of lipid peroxidation reactions. In the setting of DIC, both the expression and activity of acyl-CoA synthetase long-chain family member 4 are significantly upregulated, leading to a substantial increase in long-chain polyunsaturated fatty acids, particularly arachidonic and adrenic acids, within membrane phospholipids [22,23]. These fatty acids serve as preferential substrates for lipid peroxidation. DOX specifically induces 15-lipoxygenase-1 expression in experimental models [24], an enzyme that directly catalyzes the peroxidation of polyunsaturated fatty acids in membrane phospholipids. The resulting lipid peroxides not only compromise membrane integrity but also activate pro-inflammatory signaling pathways, such as the mitogen-activated protein kinase cascade, establishing a self-reinforcing cycle of cellular injury.

A study suggests that under the metabolic stress associated with DIC, methionine metabolism may be aberrantly rerouted, with its downstream product, S-adenosylmethionine, unexpectedly driving pro-ferroptotic metabolic fluxes [25]. Further complexity arises from the context-dependent roles of molecules that are typically cytoprotective at low levels. For example, heme oxygenase-1, when excessively expressed in DIC, may shift from a protective factor to a promoter of ferroptosis by catalyzing extensive heme degradation and releasing large amounts of free iron [26].

### 2.4 Interplay of Cell Death Pathways

Both the initiation and progression of ferroptosis in DIC are orchestrated by a highly integrated network that links multiple regulated cell-death modalities with systemic stress responses. The mitochondrion serves as a central hub and signal amplifier within this network, coupling diverse death pathways. DOX-driven mitochondrial iron accumulation, excessive reactive oxygen species generation, and lipid peroxidation directly impair mitochondrial structure and function. Mitochondrial DNA released from damaged organelles can subsequently activate cytosolic pattern-recognition receptors, such as the absent in melanoma 2 inflammasome, promoting assembly of the PANoptosome complex. This complex can trigger pyroptosis, apoptosis, and necroptosis concurrently, explaining the pronounced synergy of multi-modal cell death observed in DIC [27].

The endoplasmic reticulum, a key sensor of cellular stress, responds to disrupted homeostasis by activating the protein kinase RNA-like endoplasmic reticulum kinase (PERK)/eukaryotic initiation factor 2α (eIF2α)/activating transcription factor 4 (ATF4)–mediated integrated stress response. Recent evidence indicates that, in the context of DIC, the protein estrogen-responsive B-box protein amplifies this pathway by facilitating ubiquitination of glucose-regulated protein 78, ultimately enhancing *Nrf2* signaling and upregulating its downstream targets SLC7A11 and GPX4. This process constitutes an intrinsic endoplasmic reticulum stress–derived protective axis against ferroptosis [28].

These intracellular events unfold within a complex multicellular cardiac microenvironment. Damage-associated signals released from ferroptotic cardiomyocytes can activate resident immune cells, including B lymphocytes, which subsequently secrete pro-inflammatory mediators and autoantibodies [29]. This establishes a self-perpetuating cycle of inflammation and cell death, highlighting DIC as a systemic pathological process in which cardiomyocyte-intrinsic death mechanisms and immune responses are tightly interconnected. Thus, effective therapeutic strategies must concurrently target both cellular and immunological dimensions of disease progression.

## 3. Mechanism-Based Therapeutic Strategies

Given that DIC arises from the intricate interplay of multiple programmed cell-death pathways, particularly ferroptosis and apoptosis, as outlined in the “Molecular Mechanisms of Ferroptosis in DIC” section, effective clinical management must extend beyond isolated, reactive supportive measures. Instead, it requires a proactive, multi-level intervention strategy closely aligned with the dynamic course of disease progression. Based on the spatiotemporal sequence of injury initiation and progression, a three-tiered, integrated defense framework comprising Primary Prevention, Secondary Intervention, and Tertiary Management is proposed (Table 1, Ref. [10,11,14,17,20,23,28,30,31,32,33,34,35,36,37,38,39,40,41,42,43,44,45,46,47,48,49]). This framework aims to systematically translate mechanistic insights from basic research into a coherent, stepwise clinical action plan.

**Table 1.** **A tiered framework for the management of DOX-induced cardiotoxicity**.

Therapeutic tier & strategic objective	Exemplary agents or interventions	Core mechanism of action & target	Development stage & key notes
Primary prevention: risk mitigation & pre-exposure intervention	Dexrazoxane	Acts as an iron chelator by preferentially binding free iron or DOX–iron complexes, suppressing hydroxyl radical formation and attenuating oxidative damage [10].	It represents the current clinical standard of care and is the only regulatory-approved prophylactic agent for anthracycline-induced cardiotoxicity; however, its clinical use remains controversial due to concerns about myelosuppression and the potential attenuation of chemotherapeutic efficacy.
	Cardiac-targeted drug delivery systems	Utilizes functionalized nanocarrier systems, such as targeted peptide nanofibers (e.g., BA-NFs [30]) or stimulus-responsive nanoparticles (e.g., ROS-sensitive liposomes DBMP [31]), to enable myocardium-specific drug accumulation and controlled release, ultimately enhancing the therapeutic index.	Preclinical/translational stage; represents a frontline approach to reduce non-specific cardiac exposure through advanced pharmaceutical engineering.
	Biomarker-guided prophylaxis	Entails the early initiation of cardioprotective therapy in patients stratified as high risk based on baseline biomarker signatures, including elevated inflammatory markers (e.g., PGLYRP1, CAMP [32]) or dysregulated sphingolipid metabolites (e.g., sphingosine-1-phosphate and ceramide [33]).	Emerging clinical concept: efficacy is contingent upon the validation of precise risk-stratification models.
	Adjunctive therapy	F-α-DDB, a novel bifendate derivative, demonstrates no additional cardiotoxicity when co-administered with epirubicin in preclinical models, suggesting its potential utility as a cardioprotective adjuvant [34]. Circulating neutrophil extracellular trap DNA functions as an early-warning biomarker, increasing before detectable declines in left ventricular ejection fraction and allowing for timely clinical intervention [35].	An emerging clinical concept involving adjuvant therapies that preserve antitumor efficacy while enabling preemptive cardioprotective intervention.
Secondary intervention: antagonism of core pathological pathways	Iron homeostasis modulators	Iron chelation and storage stabilization: 5-Oxoproline (promotes GSH synthesis) [36]; protosappanin A (binds and stabilizes ferritin heavy chain) [11]. Iron efflux promotion: asiatic acid (activates *Nrf2* signaling to upregulate ferroportin) [14].	Predominantly preclinical; directly targets the initiating event of ferroptosis—intracellular iron overload.
	Antioxidant defense enhancers	GPX4 activation: selenomethionine [37]; FSP1/CoQ10 axis stabilization: idebenone (inhibits FSP1 ubiquitination and degradation) [17]; *Nrf2* pathway activation: kaempferol [38], dioscin [23]; non-canonical GPX4 upregulation: ophiopogonin D (restores β-catenin/GPX4 signaling) [39].	Early clinical exploration; aims to restore or augment endogenous cellular capacity to counteract lipid peroxidation via multiple pathways.
	Lipid peroxidation inhibitors	Key enzyme inhibition: protosappanin A (inhibits ACSL4) [11]; enzyme-mimetic antioxidants: nanozymes with GPX4-mimetic activity [40].	Preclinical stage; acts on the terminal common pathway of ferroptosis execution.
	Multi-target/pan-cell death inhibitors	BRD4770: concurrently inhibits ferroptotic and apoptotic signaling pathways [31]; EBBP: activates the PERK/eIF2α/ATF4 ISR, leading to Nrf2/SLC7A11/GPX4 upregulation [28]; dipyridamole: suppresses ferroptosis by upregulating the cystine/glutamate antiporter subunit SLC7A11 [41].	Distinctive intervention strategy; addresses crosstalk between programmed cell death pathways, potentially yielding synergistic effects.
	Dynamic metabolic biomarkers	Lycopene intervention: reversal of DOX-induced shifts in GSH (depletion) and MDA (elevation) validates these as dynamic, mechanism-based biomarkers for monitoring ferroptosis and treatment response [42].	Preclinical/translational utility; bridges therapeutic intervention with objective pharmacodynamic assessment.
Tertiary management: systemic regulation & long-term rehabilitation	Agonists of endogenous cardioprotective pathways	PGE_2_ receptor 1 activation: triggers the Gαq–PKC–*Nrf2* signaling cascade [20]; specialized pro-resolving mediators: maresin1 exerts protection via the NRF2/GPX4 axis [43].	Preclinical proof-of-concept; leverages innate cellular defence systems with a favourable side-effect profile.
	Multi-component systemic modulators	Herbal formulations: qishen granule, Xin-Ji-Er-Kang, which synergistically upregulate core defence pathways like Nrf2 through multi-target networks [44,45].	Clinical applications of some formulations embody a strategy of systemic modulation to ameliorate pathological states.
	Non-pharmacological physiological interventions	Structured endurance exercise: mitigates ferroptosis and mitochondrial dysfunction via mechanisms such as AMPKα2 activation, enhancing functional cardiac reserve [46].	Foundational supportive therapy: a safe and accessible intervention integral to cardiac rehabilitation.
	Adjunctive cardiac supportive therapies	Dexmedetomidine promotes nuclear translocation of *Nrf2* by activating the *AKT/GSK3β* signaling axis while preserving antitumor efficacy [47]; neuroendocrine antagonists, including renin–angiotensin system inhibitors and β-blockers, have demonstrated efficacy in attenuating ventricular dysfunction [48,49] and constitute standard therapeutic agents for the management of symptomatic heart failure.	Clinical adjuncts include both emerging supportive agents and guideline-directed medical therapy.

Abbreviations: DOX, doxorubicin; BA-NFs, baicalin nanofibers; ROS, reactive oxygen species; DBMP, a ROS-responsive nanoliposome containing BRD4770 and a cardiac-targeting peptide; GSH, glutathione; Nrf2, nuclear factor erythroid 2–related factor 2; GPX4, glutathione peroxidase 4; FSP1, ferroptosis suppressor protein 1; CoQ10, coenzyme Q10; ACSL4, acyl-CoA synthetase long-chain family member 4; BRD4770, a histone methyltransferase inhibitor; EBBP, estrogen-responsive B-box protein; ISR, integrated stress response; SLC7A11, solute carrier family 7 member 11; MDA, malondialdehyde; PGE_2_, prostaglandin E2; AMPKα2, adenosine monophosphate-activated protein kinase alpha2; PGLYRP1, peptidoglycan recognition protein 1; CAMP, cathelicidin antimicrobial peptide; F-α-DDB, a novel synthetic derivative of bifendate; eIF2α, eukaryotic initiation factor 2α; PKC, Protein kinase C; AKT, protein kinase B; GSK3β, Glycogen Synthase Kinase 3β.

### 3.1 Primary Prevention

This tier prioritizes preemptive intervention and offers the greatest clinical and economic value by preventing cardiac injury before its onset. Ongoing controversies surrounding dexrazoxane, particularly concerns related to myelosuppression and potential compromise of antitumor efficacy, highlight the pressing need for alternative prophylactic strategies. As a result, two major directions have emerged: advanced cardiac-targeted drug delivery systems that reduce myocardial exposure through pharmaceutical engineering, and biomarker-guided precision prophylaxis. The latter approach applies baseline risk stratification, such as elevated inflammatory markers including peptidoglycan recognition protein 1 (PGLYRP1) [32], to identify patients at high risk who may benefit from early intervention. Promising adjuvant agents, exemplified by a novel synthetic derivative of bifendate (F-α-DDB) [34], which did not exacerbate epirubicin-induced cardiotoxicity in preclinical studies, reflect efforts to achieve cardioprotection without diminishing chemotherapeutic effectiveness.

### 3.2 Secondary Intervention

This tier represents the central therapeutic response, directly targeting the molecular effectors responsible for cardiotoxic injury. Its structure aligns with the core ferroptosis cascade, encompassing dysregulated iron metabolism, failure of antioxidant defenses, and lethal lipid peroxidation. Similarly, therapeutic agents are designed to interrupt this process at key control points, ranging from iron chelators and iron efflux enhancers (e.g., asiatic acid [14]) to antioxidants that directly reinforce GPX4 activity (e.g., selenomethionine [37]). This tier also incorporates emerging regulatory nodes, such as the estrogen-responsive B-box protein [28], which suppresses ferroptosis by activating the integrated stress response, as well as multi-target compounds like a histone methyltransferase inhibitor (BRD4770) [31] that concurrently inhibit ferroptosis and apoptosis. Such agents may offer advantages in counteracting the interconnected PANoptotic death network.

### 3.3 Tertiary Management

Once cardiotoxic injury has occurred, therapeutic priorities shift from limiting cell death to enhancing endogenous repair mechanisms, systemic resilience, and long-term functional recovery. This tier capitalizes on intrinsic homeostatic capacity and includes pharmacological activation of endogenous protective pathways, such as the prostaglandin E2 (PGE_2_)/EP1 signaling axis [20], as well as multi-component systemic modulators, including traditional herbal formulations like qishen granules [44], which exert effects through pleiotropic regulatory networks. Foundational non-pharmacological strategies play a critical role. Structured exercise programs [46], for example, represent a highly accessible and effective intervention that improves mitochondrial function, attenuates ferroptosis, and serves as a cornerstone of comprehensive cardiac rehabilitation.

Effective management of DIC requires a dynamic, coordinated implementation of a multi-tiered strategy. This process begins with pre-treatment risk stratification based on genetic profiles and baseline biomarkers, which should inform subsequent preemptive interventions focused on source control, such as cardiac-targeted drug delivery systems or prophylactic agents in high-risk patients. This preventive foundation must be integrated with close monitoring throughout chemotherapy, using sensitive modalities including global longitudinal strain and emerging mechanism-specific biomarkers, such as circulating neutrophil extracellular trap DNA [35], to identify subclinical cardiac injury at the earliest stage. Early detection should prompt timely initiation of mechanism-directed therapies that directly inhibit core pathological processes, particularly ferroptosis. Throughout treatment and into survivorship, this strategy should be reinforced by adjunctive systemic modulation and structured rehabilitation, including exercise and selected natural compounds, to enhance endogenous repair capacity, preserve functional reserve, and improve long-term cardiac outcomes. Translating this comprehensive framework into routine clinical practice remains a major challenge, requiring sustained advances in translational research, rigorous validation of personalized monitoring strategies, and careful optimization to ensure cardioprotection without compromising antitumor efficacy.

## 4. Integrated Surveillance Across the Clinical Continuum

DIC typically progresses along a defined continuum, beginning with subclinical myocardial injury, advancing to asymptomatic left ventricular dysfunction (ALVD), and potentially evolving into overt heart failure. Evidence indicates that failure to detect and manage ALVD substantially increases long-term mortality risk [50]. As a result, establishing a dynamic monitoring system that encompasses the full continuum of “risk prediction–early detection–treatment response assessment–long-term follow-up” is essential for effective cardioprotection. Conventional surveillance strategies based primarily on left ventricular ejection fraction (LVEF) and cardiac troponin levels are often insufficient to capture early, potentially reversible myocardial injury [51]. Therefore, this section integrates established clinical assessments with emerging mechanism-driven biomarkers, advanced imaging modalities, and digital health technologies to propose a hierarchical, interconnected, and comprehensive monitoring framework.

### 4.1 Risk Stratification and Baseline Profiling

Accurate monitoring begins with rigorous pre-treatment risk stratification. Current clinical practice largely adheres to international guidelines that classify risk based on conventional parameters, such as cumulative DOX dose (≥250 mg/m^2^ considered high risk) and patient age [52]. However, these factors alone do not adequately account for the significant interindividual variability in cardiotoxic risk observed under comparable treatment exposures.

Genetic susceptibility constitutes a critical complementary dimension of risk assessment. Pharmacogenomic studies have demonstrated that specific genetic polymorphisms influence DOX metabolism, cellular transport, and cardiomyocyte repair capacity. Variants in genes such as retinoic acid receptor gamma and solute carrier family 28 member 3 have been clearly linked to increased susceptibility to anthracycline-induced cardiotoxicity [53]. Polymorphisms in cytochrome P450 enzymes and ATP-binding cassette subfamily B member 1 modulate drug pharmacokinetics, altering myocardial exposure to toxic metabolites [54]. Incorporating genetic risk profiling before treatment initiation can therefore more precisely identify patients who require intensified surveillance and early intervention, beyond what traditional clinical factors can achieve.

Baseline circulating biomarkers further enhance predictive accuracy. Growing clinical evidence indicates that serum biomarker profiles obtained before chemotherapy can reliably predict subsequent cardiotoxic risk. For instance, a prospective study in breast cancer patients demonstrated that, even before the first chemotherapy cycle, individuals who later developed cardiotoxicity showed significantly elevated mRNA and protein levels of four biomarkers, PGLYRP1, cathelicidin antimicrobial peptide (CAMP), matrix metallopeptidase 9 (MMP9), and carcinoembryonic antigen-related cell adhesion molecule 8 (CEACAM8), in peripheral blood compared with those who remained unaffected [32]. Similarly, a retrospective analysis revealed that baseline serum concentrations of sphingosine-1-phosphate and dihydrosphingosine-1-phosphate, as well as plasma levels of specific ceramides measured before anthracycline-based therapy, were significantly associated with adverse cardiac outcomes following treatment [33]. These results suggest that pre-existing or chemotherapy-unmasked subclinical inflammatory and metabolic states serve as important biological indicators of cardiac vulnerability.

### 4.2 Dynamic Surveillance for Subclinical Injury

The treatment phase represents a critical window for detecting and intervening in reversible subclinical myocardial injury, particularly ALVD, necessitating monitoring tools with greater sensitivity than LVEF. Circulating biomarker panels offer promising opportunities for early detection. Conventional markers of myocardial injury, such as high-sensitivity cardiac troponin and N-terminal pro–B-type natriuretic peptide, while highly specific, show limited sensitivity (approximately 8%–32%) for identifying ALVD [50] and therefore primarily reflect established injury rather than early dysfunction. Biomarkers that directly capture ferroptosis-related pathology provide a novel dimension for early surveillance. For example, reductions in serum GSH and elevations in malondialdehyde directly indicate a collapse of intracellular antioxidant defenses and ongoing lipid peroxidation [42]. Furthermore, alterations in proteins associated with regulatory pathways, such as the angiotensin II type 1 receptor and the fat mass and obesity-associated protein/p21 axis, reflect upstream signaling disturbances linked to cardiotoxicity [30,55]. Longitudinal monitoring of these mechanistic biomarkers may enable earlier identification of cardiotoxic initiation and progression than traditional indicators.

Cardiac imaging modalities are advancing toward greater sensitivity and quantitative precision. Echocardiographic global longitudinal strain (GLS) is well established as a more sensitive marker of subclinical myocardial dysfunction than LVEF, with early GLS impairment strongly associated with the subsequent development of overt cardiac dysfunction [56]. Cardiac magnetic resonance imaging, further enhances early detection through techniques such as longitudinal relaxation time mapping, extracellular volume quantification, and transverse relaxation time mapping, which non-invasively assess myocardial fibrosis and edema and can reveal histopathological changes concurrent with or even preceding GLS abnormalities. Moreover, emerging molecular imaging probes have enabled *in vivo*, ferroptosis-specific imaging in animal models, detecting myocardial injury signals 24–48 hours earlier than conventional modalities [57]. Although currently confined to preclinical research, these approaches highlight the potential for ultra-early and mechanism-specific diagnosis in the future.

The integration of intelligent technologies and digital health platforms is further transforming the paradigms of cardiac monitoring. Deep learning–based artificial intelligence models can synthesize multi-modal data, including echocardiographic parameters (e.g., tissue Doppler imaging), longitudinal clinical variables, and biomarker profiles, to generate comprehensive risk assessments. Such models have demonstrated high accuracy in predicting medium-term (e.g., 24-month) risk of cancer therapy–related cardiac dysfunction and can identify subtle patterns that may elude human interpretation [58]. Similarly, wearable devices enabling continuous remote monitoring of heart rate, rhythm, and patient-reported outcomes, such as exercise intolerance and dyspnea, can capture early compensatory changes or emerging symptoms between clinic visits. When integrated into clinical decision support systems, these data streams can automatically trigger alerts, shifting cardiac surveillance from intermittent, clinic-based evaluations to continuous, dynamic, whole-course management [59].

### 4.3 Post-Treatment Long-Term Management

The final stage of monitoring focuses on objectively evaluating therapeutic effectiveness while proactively addressing the persistently elevated cardiovascular risk associated with DOX exposure. When cardioprotective interventions, such as ferroptosis inhibitors, are initiated in response to monitoring findings, objective and highly sensitive metrics are required to assess treatment efficacy. Biomarkers closely linked to the underlying injury mechanisms, including the GSH-to-malondialdehyde ratio or the magnitude of improvement in GLS, can serve as early and responsive indicators of therapeutic benefit, informing timely treatment adjustment and optimization [60].

DOX-induced cardiotoxicity is characterized by delayed onset, with clinical manifestations potentially emerging years or even decades after completion of chemotherapy. Therefore, long-term cancer survivors should be incorporated into structured cardiovascular surveillance programs that include regular evaluation of cardiac function, such as GLS and LVEF, alongside rigorous management of conventional cardiovascular risk factors. This longitudinal care should be closely integrated with patient-reported outcomes and remote monitoring technologies, establishing an active, continuous system for early detection and prevention of symptomatic heart failure and premature atherosclerotic cardiovascular disease [59].

## 5. Conclusions and Future Perspectives

Ferroptosis has been established as a central pathogenic mechanism in DIC. Its activation arises from a synergistic cascade in which DOX disrupts cardiomyocyte iron homeostasis, compromises the GPX4/Nrf2-centered antioxidant defense system, and induces lipid metabolic reprogramming, culminating in lethal lipid peroxidation. To date, the principal pathways governing iron metabolism (e.g., *NCOA4*-mediated ferritinophagy), antioxidant defense, and lipid peroxidation have mainly been delineated. However, critical knowledge gaps remain, particularly regarding cell-type–specific regulatory mechanisms, dynamic crosstalk among distinct programmed cell-death pathways, and the integrative role of organelle homeostasis in shaping ferroptotic outcomes.

From a therapeutic perspective, intervention strategies are transitioning from isolated approaches toward an integrated framework encompassing “prevention–antagonism–repair”. Advances in targeted drug delivery and precision risk stratification have strengthened preventive capacity, while inhibitors directed at key ferroptotic nodes have shown encouraging efficacy in preclinical models. Systemic repair strategies, including activation of endogenous cardioprotective pathways, are attracting increasing interest. However, most emerging agents remain at the preclinical stage, and their cardiac specificity, long-term safety, and potential effects on antitumor efficacy require rigorous validation. Moreover, therapies capable of simultaneously modulating multiple cell-death pathways are still in their infancy.

Management paradigms are shifting away from reliance on delayed indicators toward early-warning systems that integrate mechanistic biomarkers, sensitive imaging modalities, and artificial intelligence–based analytics. Despite this progress, an optimal implementation pathway has yet to be defined. Major challenges include the absence of standardized ferroptosis-specific circulating biomarkers, the limited maturity of individualized risk prediction models, and the difficulty of seamlessly integrating novel monitoring and intervention strategies with established clinical tools and rehabilitation programs into cohesive, whole-course management frameworks.

Future progress will require coordinated advances across three interdependent domains: mechanisms, strategies, and management. Spatial omics and related high-resolution technologies should be leveraged to delineate the cell-type–specific ferroptosis landscape throughout disease progression and to unravel its network-level regulation. Promising targeted delivery platforms should be advanced into clinical investigation, alongside biomarker-guided prospective trials to validate precision-based preventive and therapeutic approaches. From a management standpoint, interdisciplinary collaboration is urgently needed to develop standardized data infrastructures, accelerate the clinical translation of biomarkers, and integrate remote monitoring and intelligent decision-support systems into routine care. By addressing these priorities, it may become possible to transform DOX-induced cardiotoxicity into a condition that is preventable, detectable at an early stage, and amenable to precise intervention, preserving long-term cardiac health without compromising oncologic efficacy.
